# Liver metastasized pancreatic neuroendocrine tumor in a 17‐year‐old female: A case report

**DOI:** 10.1002/ccr3.9545

**Published:** 2024-11-04

**Authors:** Mahmoud Sultan, Azad Al Hasan, Nisreen Khazem

**Affiliations:** ^1^ Faculty of Medicine Damascus University Damascus Syria

**Keywords:** endocrinology and metabolic disorders, gastroenterology/hepatology, oncology, pathology and laboratory medicine, pediatrics and adolescent medicine

## Abstract

**Key Clinical Message:**

Pancreatic neuroendocrine tumors (PNETs) are rare and often misdiagnosed due to their vague symptoms and tumor heterogeneity. Early detection using computed tomography (CT) is essential, particularly in regions without access to advanced diagnostic tools like immunohistochemistry and genetic testing.

**Abstract:**

Neuroendocrine tumors (NETs) are rare tumors in adults and extremely rare in the pediatric population, as pancreatic NETs (pNETs) have an incidence rate of <0.1 per million. We present a case of a 17‐year‐old female with a liver metastasized pNET. A 17‐year‐old female with a history of intermittent abdomen‐back pain presented to the clinic with severe upper abdominal pain radiating to the shoulder. The routine tests were normal. An ultrasound showed multiple lesions in the liver, which were confirmed by a computed tomography (CT) that uncovered a pancreatic lesion too. A liver biopsy proved it was a metastasized pNET with positive NET markers on IMC staining. The metaiodobenzylguanidine (MIBG) scan flared the liver lesion. The patient was started on Octreotide long‐acting release (LAR) 30 mg once monthly. The rarity of these tumors makes their diagnosis difficult, but they should not be omitted and must be considered when there are long‐lasting symptoms that are not compatible with common illnesses. These tumors are curable in their early stages.

## 1 INTRODUCTION

Neuroendocrine tumors (NETs) originate from the neuroendocrine cells dispersing throughout the body tissues and organs, making them potentially malignant due to their ability to metastasize.^1^ Metastasizing ability is concerning, especially considering the wide range of tissues it can affect in adults. While less common in children, NETs can develop in nearly any pediatric tissue. Interestingly, gastroenteropancreatic NETs are particularly rare in this age group, with an estimated incidence of 2.8 cases per million.^2^ Despite their overall rarity, their incidence seems to have been gradually rising in the past few decades.^3^, ^4^ This increase could be a result of improved diagnostic techniques leading to more disease detection or might reflect a real augmentation of onset.^5^ Consequently, NETs can manifest almost anywhere, particularly in the gastrointestinal tract and lungs.^1^ Pancreatic NETs (PNETs), originating from the islets of Langerhans, are particularly uncommon, representing less than 2% of digestive tract tumors and less than 1% of all NETs. They primarily occur between the 4th and 6th decades of life but can develop at any age.^6^, ^7^ With an incidence rate of less than 0.1 per million, PNETs are exceptionally rare.^8^ Further complicating diagnosis, PNETs often exhibit slow, unpredictable growth and produce non‐specific symptoms, leading to missed diagnoses and delayed curative treatment.^9^ We exemplify these challenges by presenting the case of a 17‐year‐old girl who has had a long history of intermittent abdominal pain until it worsened and was diagnosed with liver metastasized pNET.

## 2 CASE PRESENTATION

### 2.1 Patient information

A 17‐year‐old female came to the clinic with somewhat severe upper abdominal pain radiating to the shoulder. The family mentioned a previous diagnosis of gastroenteritis and renal colic managed by drugs prescribed by an outside local clinic. No surgical or allergy history. The familial history was also negative.

### 2.2 Timeline

The pain started around 2 months ago, awakening her from sleep, partially responsive to analgesics, worsening on supination, and not related to eating. There was a history of similar intermittent, non‐specific abdomen and back pain throughout the past few years, which was responding to analgesics and anti‐spasms. She mentioned nausea and loss of appetite, which were fixed by a drug called Cypro‐Vita™, and denied loss of weight, persistent diarrhea, or any flushing.

### 2.3 Clinical findings

The clinical examination was insignificant except for tenderness in the upper abdomen.

### 2.4 Diagnostic assessment

**FIGURE 1:**
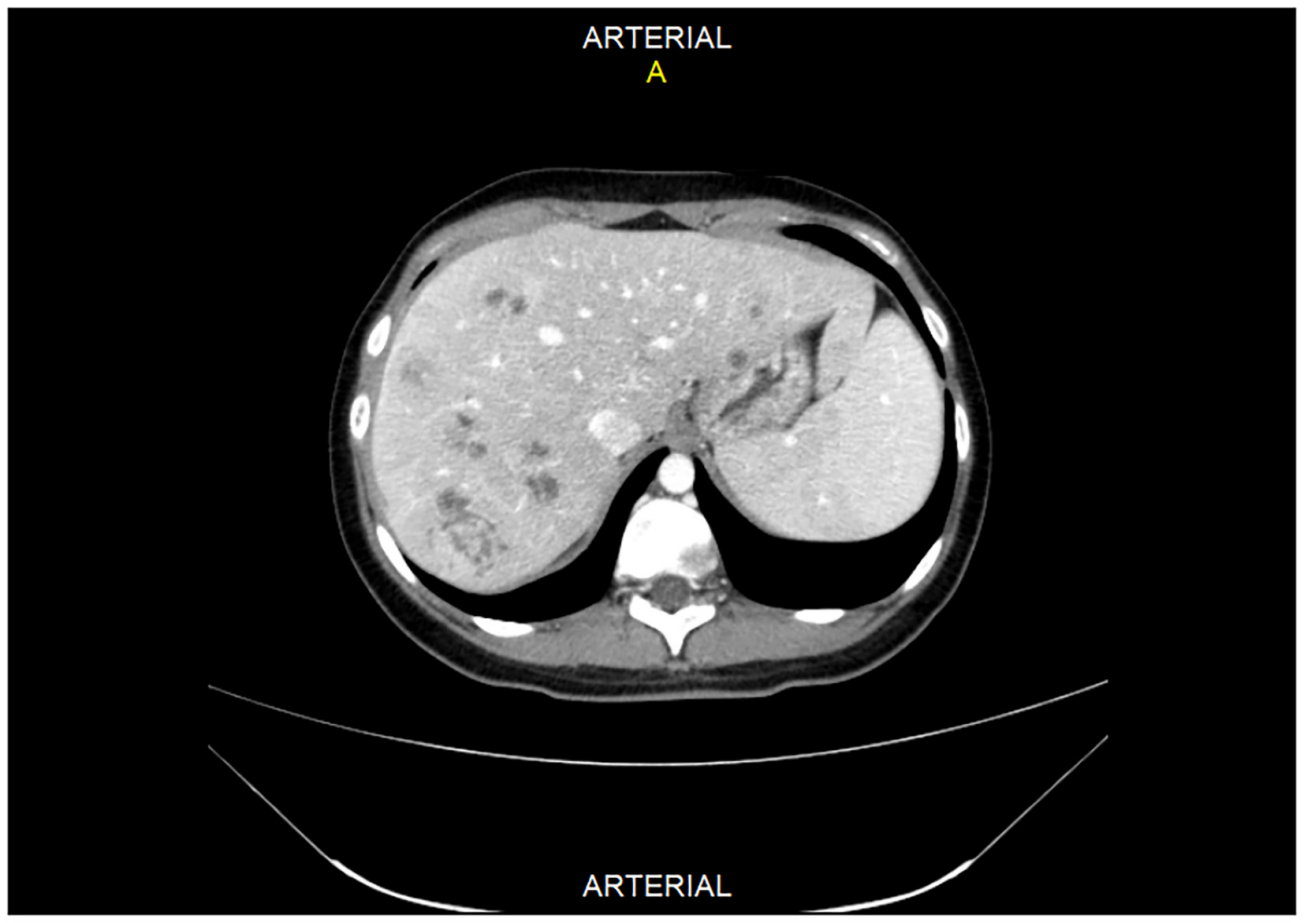
A 17‐year‐old female with metastasized pancreatic neuroendocrine tumor. CT image of the liver metastases in the arterial phase. Showing multiple well‐defined heterogeneous masses in the liver hemi‐lobes.

**FIGURE 2:**
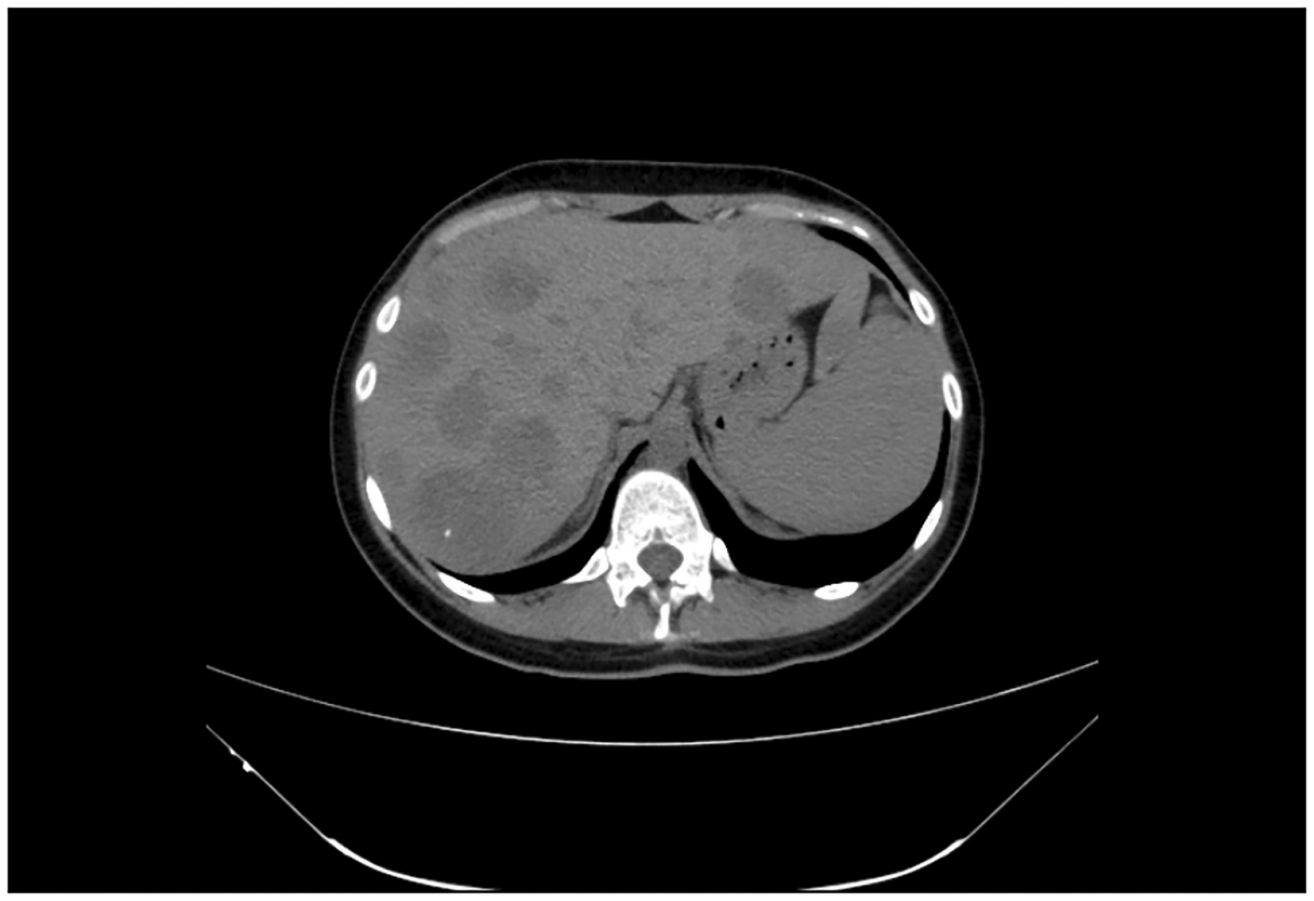
A 17‐year‐old female with metastasized pancreatic neuroendocrine tumor. CT image of the liver metastases in the plain phase. Showing multiple well‐defined heterogeneous masses in the liver hemi‐lobes.

**FIGURE 3:**
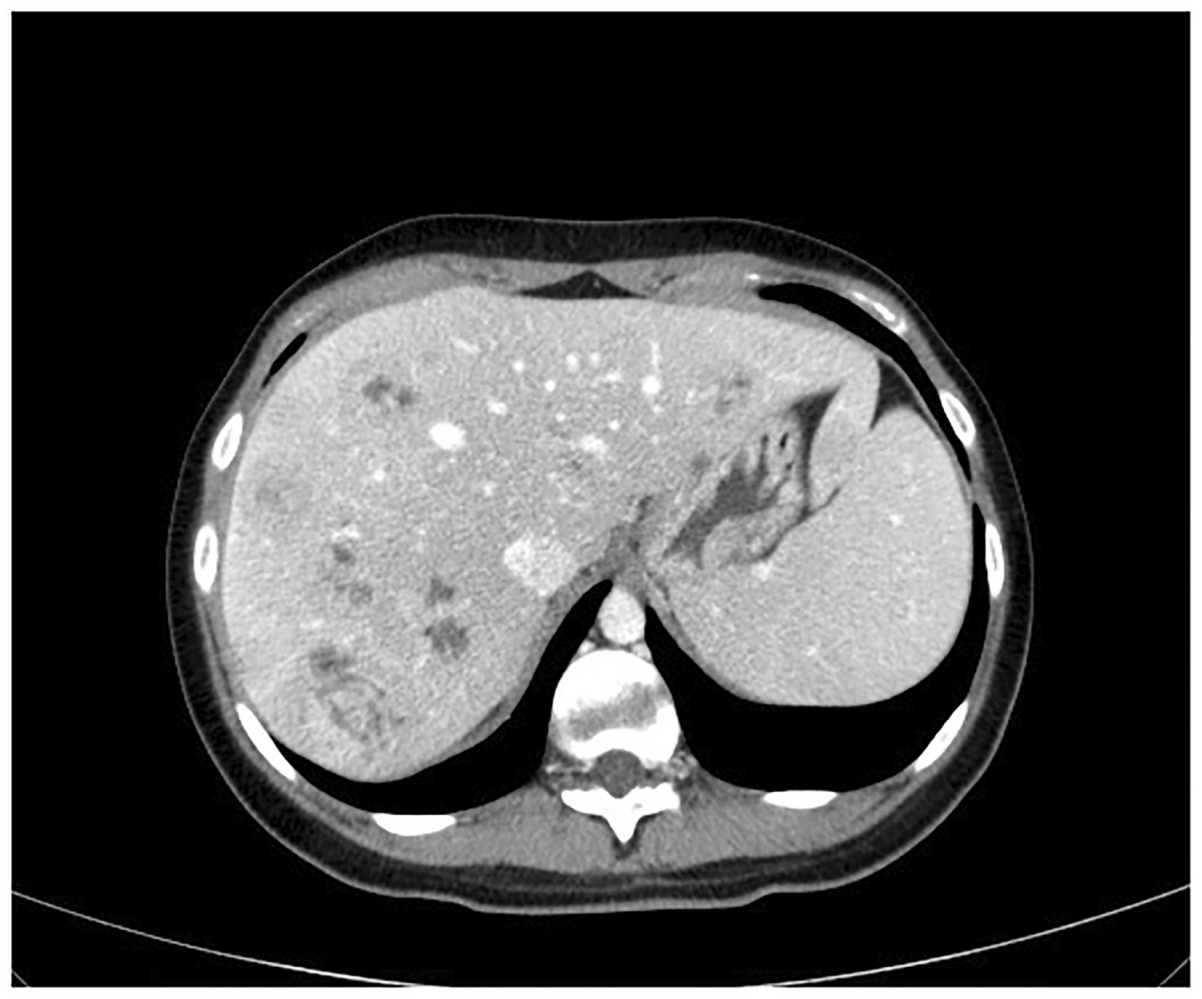
A 17‐year‐old female with metastasized pancreatic neuroendocrine tumor. CT image of the liver metastases in the portal phase. Showing multiple well‐defined heterogeneous masses in the liver hemi‐lobes.

**FIGURE 4:**
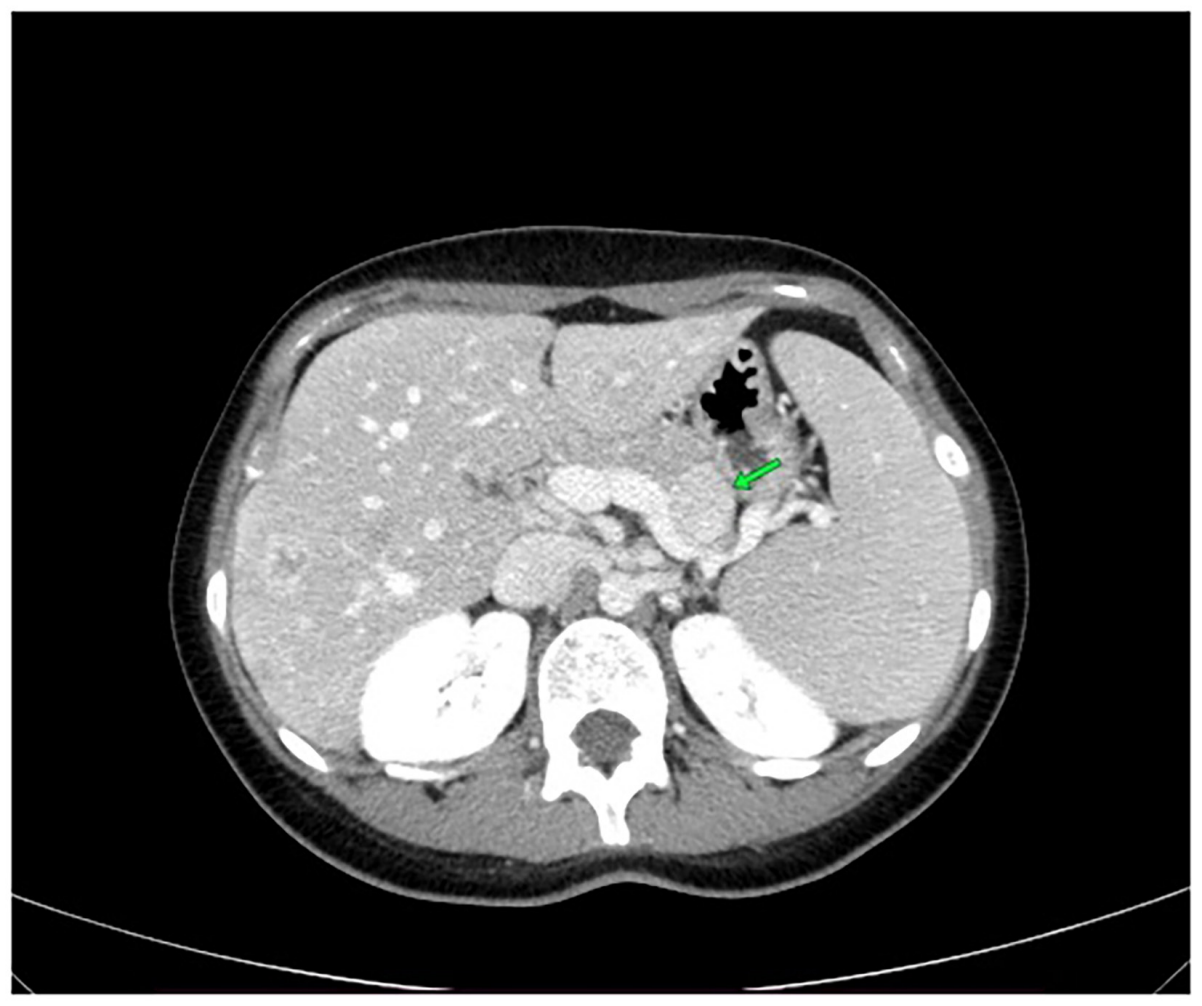
A 17‐year‐old female with metastasized pancreatic neuroendocrine tumor. CT image of the pancreatic mass showing a well‐defined heterogeneous mass in the tail.

**FIGURE 5:**
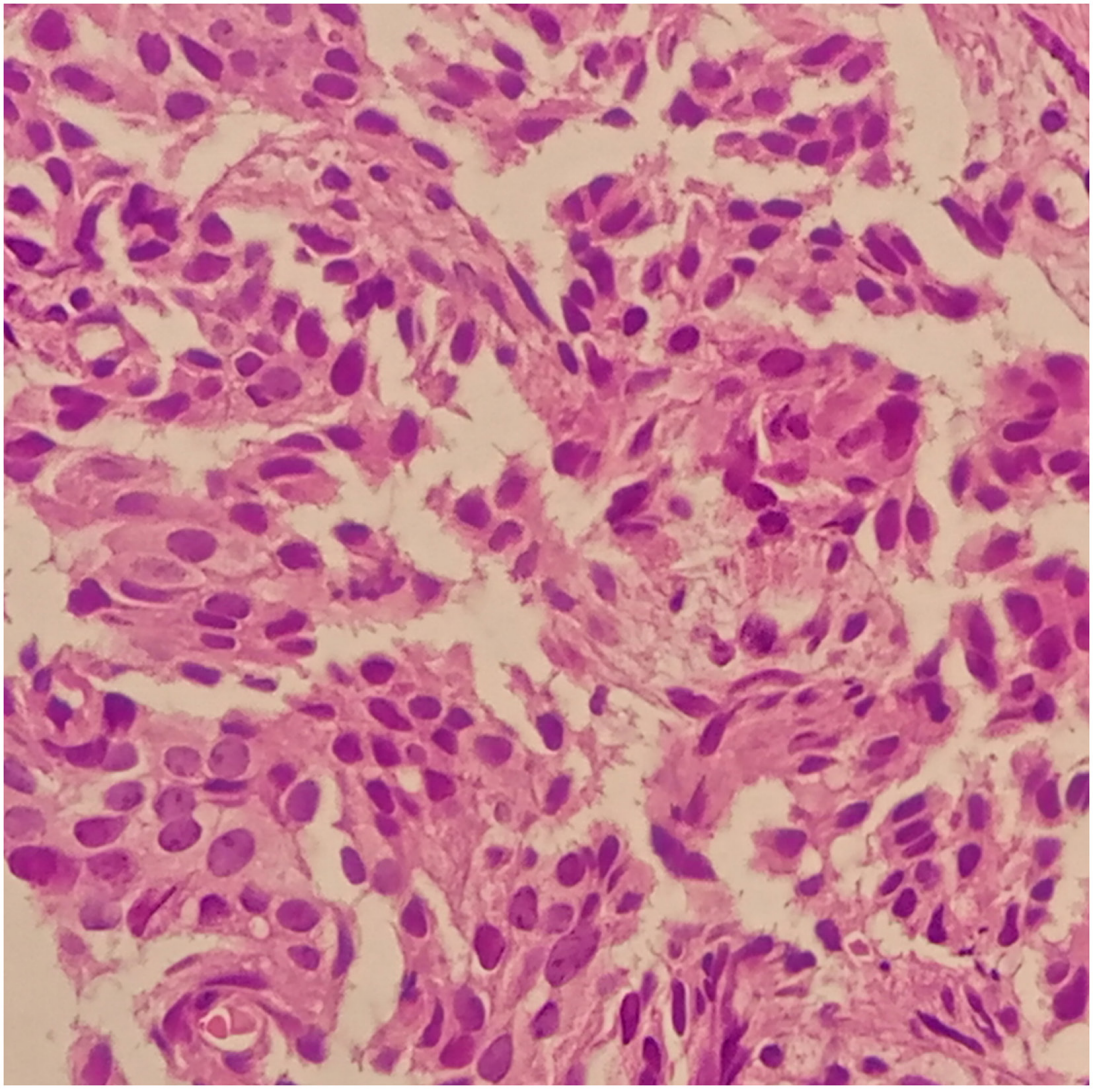
The pathological findings of the liver mass biopsy.

**FIGURE 6:**
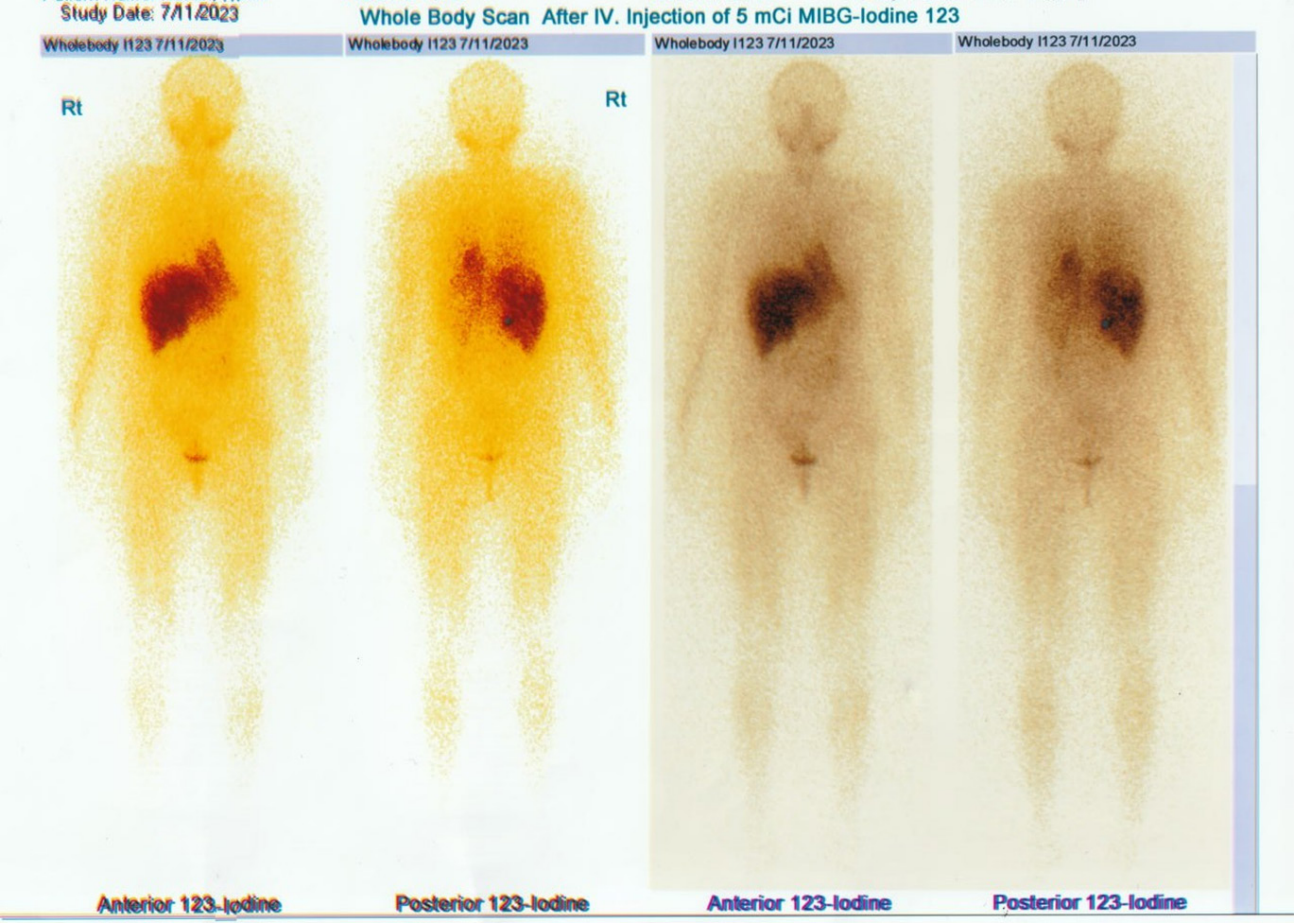
The MIBG scan image, showing a pathologic focus of the radioactive material 123I in the liver.

The ultrasound revealed multiple hyper‐echoic lesions with a hypo‐echoic surrounding area in the liver hemi‐lobes with no extending of the biliary tract inside and outside. Blood workup and liver and kidney functionality were within normal ranges, as were the values of prolactin, thyroid releasing hormone (TRH), growth hormone (GH), and parathyroid hormone (PTH). She was scheduled for both upper and lower endoscopies, which showed no abnormal findings. The breast ultrasound too revealed no abnormalities. The computed tomography (CT) with contrast showed multiple hypo‐dense masses disseminated through the liver that have a peripheral fixation of the contrast (Figures 1, 2, 3, 4), ranging from 10 to 40 mm, constituting 50% of the liver, and a blurred hypo‐dense lesion between the pancreatic body and tail measuring around 25 mm. Then an endoscopic ultrasound (EUS) was performed. The pancreas had a white streak appearance with some reactive lymph nodes around it, but no recognizable mass was seen. Following the EUS, a transcutaneous liver biopsy was performed. The biopsy results revealed a well‐differentiated neuroendocrine tumor. Given the presence of the pancreatic lesion, this was diagnosed as a metastatic pancreatic NET (Figure 5). The immunohistochemical staining showed positivity for chromogranin, synaptophysin, CD56, CK (Cytokeratin) and a ki‐67 index of 5%. Laboratory results of chromogranin A (CgA), NSE (neuron‐specific enolase), and 5‐HIAA (5‐hydroxyindoleacetic acid) were: 1499 ng/dL (reference range being: 0–100), 32.3 ng/dL (0–17), and 9.30 mg/24 h (2–10), respectively. An 123I‐meta‐iodobenzylguanidine (123I‐MIBG) scan confirmed the liver metastasis (Figure 6).

### 2.5 Therapeutic intervention

The patient was started on Octreotide LAR 30 mg once every month.

### 2.6 Follow‐up and outcomes

A follow‐up appointment was scheduled for 6 months later.

## 3 DISCUSSION

PNETs form two groups: functional pNETs and non‐functional pNETs. A‐pNETs secrete hormones (common are insulinoma, gastrinoma) actively causing related symptoms and thus leading to an early diagnosis.^6^ While NA‐pNETs may release some substances like chromogranin, synaptophysin, NSE, and ghrelin. These secretions do not cause symptoms specific to them, which makes it challenging for physicians to suspect their oversecretion and trace their source tumors in the beginning before progressing any further. Rather they tend to manifest much later with abdominal pain, weight loss, anorexia, and nausea. Of course, by then in the disease course, these indefinite symptoms can be caused by metastasis itself, which usually occurs in the liver or the bones (between 32% and 73% of cases are metastatic at diagnosis). The majority of NENs are sporadic, but hereditary syndromes that predispose to them are multiple endocrine neoplasia type 1 syndrome (MEN‐1)/Wermer syndrome type 1, Von Hippel–Lindau syndrome, neurofibromatosis type 1, and tuberous sclerosis.^7^, ^10^ with the prolactin and parathyroid hormone (PTH) tests being within the normal range, indicating non‐existence of secreting prolactinoma or hyperparathyroidism, and with that we rendered Wermer syndrome to be unlikely in this setting, although we were not able to fully rule it out by genetic testing, which was not done in our case.

It is crucial to pay attention to the differential diagnosis of PNETs and distinguish them as the treatment options differ widely. Differential diagnoses of PNETs include pancreatic ductal adenocarcinoma (PDAC), chronic mass formatting pancreatitis (CMFP), and intrapancreatic accessory spleen (IPAS). CT scans play an essential role in distinguishing between them in a preoperative manner. while PDAC and CMFP share similar imaging findings with hypovascular pancreatic neuroendocrine tumors (hypo‐PNETs), IPAS mimics findings with hypervascular pancreatic neuroendocrine tumors (PNETs).^11^, ^12^, ^13^ When compared to PDAC, hypo‐PNETs at CT imaging displayed lower frequencies of local invasion or metastasis and higher frequencies of a well‐defined margin. Compared to PDAC, the mean attenuation of PNETs at the arterial and portal venous phases was noticeably higher. In particular, the degree of amplification at the arterial phases could be helpful in separating PNETs from PDACs using CE‐CT.^11^ In contrast to CMFP, hypo‐PNETs were less likely to have a history of pancreatitis and calcification and more likely to have a well‐defined margin and cystic alterations. In the portal and delayed phases, CMFP exhibited greater mass contrast enhancement and mass‐to‐pancreas enhancement ratio than hypo‐PNETs.^12^ Additionally, we can rely on the absolute apparent diffusion coefficient (ADC) and normalized ADC (lesion‐to‐spleen ADC ratios) to clinically distinguish between IPAS and hyper‐PNETs. In comparison to hyper‐PNETs, IPAS exhibited notably lower absolute ADC and normalized ADC values.^13^

Although the newly developed PET/CT (positron emission tomography) with radiolabeled somatostatin analogs such as 68 Ga‐DOTATATE has been suggested as the new gold standard and the first‐line diagnostic tool in both adults and children,^1^, ^14^ due to its unavailability, it was not applied.

One of the noticeable findings in our case is the high level of serum CgA, which is assumed to be correlated with liver metastases, as shown in several studies.^15^ With the liver metastasis, the tumor's stage is considered IV, applying the AJCC 8th (American Joint Committee on Cancer) and European Neuroendocrine Tumor Society (ENETS) staging classification.^16^ and by the histopathologic grading classifications of World Health Organization (WHO) 2017/2019, it would fall within the Grade 2 (G2) standards.^16^ On the prima facie that NETs have an increased somatostatin receptor expression, we started the patient on the targeted therapy Octreotide LAR 30 mg, a long‐active‐release somatostatin analog that, along with its symptoms relieving characteristics, has shown antiproliferative and cytostatic effects.^17^

The extremely rare existence of this tumor in ages like our patient might have made the physicians omit it from the differential diagnosis of her symptoms, but the long history of the intermittent abdomen, concomitant with back pain, which can be, from a retrospective view, connected to pancreatic origin, should have raised suspicion toward some indolent pancreatic lesion. But in our case, the pain was repeatedly attributed to common and non‐specific illnesses and treated as such. That led to a major delay in diagnosis as a result of the low index of suspicion surrounding these tumors. And therefore, the surgical approach, curative in early‐discovered local tumors,^18^ could not have been used since the tumor was deemed unresectable, owing to the widespread of metastases in the liver. This might have been avoided if there were a higher level of suspicion and more tests were ordered. In cases like ours, we encourage physicians to follow‐up on the symptoms in order to identify these tumors in their early stages and provide a chance of optimal recovery for our patients, evading the remarkable effect of these slowly‐progressing diseases on patient life expectancy. Moreover, this report seeks to highlight the crucial role of research and innovation in advancing the field of oncology, especially concerning rare and aggressive tumors.

### 3.1 Limitations

No genetic tests or analyses were done in our case. Moreover, There were no specific IHCs supporting the pancreatic origin in our diagnosed NET. No double balloon endoscopy was performed.

## 4 CONCLUSION

PNETs are a heterogeneous group of tumors that originate from neuroendocrine cells, and their incidence has been increasing annually. Due to PNETs' rarity, tumor heterogeneity, distinct indolent biology, and vague clinical symptoms, an appropriate early diagnosis may be missed or delayed. PNETs are currently more common than pancreatic ductal adenocarcinoma because of their indolent nature, which makes a deeper understanding of them even more important and pertinent. Furthermore, differentiating PNETs from other comparable neoplasms with CT can be crucial in developing nations where particular diagnostic procedures, including the specific IHCs for PNETs and genetic tests, are not available.

## AUTHOR CONTRIBUTIONS

**Mahmoud Sultan:** Investigation; methodology; resources; software; writing – original draft; writing – review and editing. **Azad Al Hasan:** Methodology; resources; writing – original draft; writing – review and editing. **Nisreen Khazem:** Conceptualization; project administration; supervision; writing – review and editing.

## FUNDING INFORMATION

None declared.

## CONSENT

A written informed consent was obtained from the patient's parents to publish this report in accordance with the journal's patient consent policy.

## RESEARCH REPORTING GUIDELINES

The authors confirm that they followed the CARE Guidelines in writing and reporting this case report.

## Data Availability

The data that support the findings of this study are available from the corresponding author upon reasonable request.
